# Repurposing Metal-Based Therapeutics for Human Metapneumovirus (HMPV): An Integrative Computational Approach

**DOI:** 10.1155/bca/6680612

**Published:** 2025-10-17

**Authors:** Amit Dubey, Manish Kumar, Aisha Tufail, Vivek Dhar Dwivedi, Andrea Ragusa

**Affiliations:** ^1^Center for Global Health Research, Saveetha Medical College and Hospitals, Saveetha Institute of Medical and Technical Sciences, Chennai, Tamil Nadu, India; ^2^Department of Biochemistry, Iswar Saran Degree College, University of Allahabad (A Constituent PG College of University of Allahabad), Prayagraj, India; ^3^Computational Chemistry and Drug Discovery Division, Quanta Calculus, Greater Noida 201310, Uttar Pradesh, India; ^4^Bioinformatics Research Division, Quanta Calculus, Greater Noida 201310, India; ^5^CNR-Nanotec, Institute of Nanotechnology, Via Monteroni, Lecce 73100, Italy; ^6^Department of Life Sciences, Health and Health Professions, Link Campus University, Via del Casale Di San Pio V 44, Rome 00165, Italy

## Abstract

Human metapneumovirus (HMPV) is a respiratory pathogen of global concern, particularly affecting infants, the elderly, and immunocompromised individuals. Despite its prevalence, no targeted antiviral therapies are currently approved. In this study, we employed a structure-guided computational strategy to repurpose clinically approved metal-based drugs as potential HMPV inhibitors. A curated chemical library was screened against the HMPV fusion protein (PDB ID: 5WB0) using high-accuracy molecular docking, followed by molecular dynamics (MD) simulations (2000 ns), binding free energy calculations, and pharmacophore modeling. Top-ranked compounds—Auranofin, silver sulfadiazine, and gallium nitrate—exhibited superior binding affinities (ΔG_binding: −68.5 to −62.7 kcal/mol), stable protein–ligand complexes (RMSD: 2.1–2.4 Å), and consistent interaction profiles when benchmarked against known antivirals ribavirin and favipiravir. Quantum chemical descriptors derived from density functional theory (DFT) and molecular electrostatic potential (MESP) mapping confirmed their favorable electronic properties, including optimal HOMO–LUMO gaps and total energy stability. Furthermore, ADMET predictions revealed acceptable oral bioavailability, low predicted toxicity, and renal clearance profiles, though known risks such as gallium accumulation were acknowledged. This integrative study highlights the potential of repurposed metallodrugs as novel anti-HMPV agents, offering a rational and cost-effective path toward therapeutic advancement.

## 1. Introduction

Human metapneumovirus (HMPV) is a significant respiratory pathogen that has garnered global attention due to its impact on both pediatric and adult populations, contributing to severe respiratory illnesses such as bronchiolitis and pneumonia [1, 2]. Recent epidemiological studies highlight the substantial role of HMPV in community-acquired pneumonia among individuals over 60 years of age, with many cases requiring hospitalization due to severe symptoms [3]. The absence of targeted therapeutic agents for HMPV poses a critical challenge to global public health systems [4].

Advances in computational methodologies have revolutionized drug discovery, offering precision and efficiency in identifying therapeutic candidates [5, 6]. These methods—encompassing molecular modeling, quantum chemistry, and bioinformatics—have enabled a paradigm shift from traditional trial-and-error approaches to mechanism-driven predictions [7]. Their adoption is particularly essential in the case of HMPV, where high viral mutability and complex host interactions hinder classical drug development pathways [8, 9].

Recent investigations have underscored the utility of in silico platforms in identifying novel inhibitors against HMPV. For instance, virtual screening and structure-based drug repurposing strategies have revealed promising antiviral candidates with dose-dependent efficacy [10]. Simultaneously, crystallographic studies of the HMPV fusion (F) protein (PDB ID: 5WB0) have provided a structural basis for rational inhibitor design [11, 12]. These advancements underscore the necessity of integrating computational workflows to expedite the identification of viable anti-HMPV agents.

In the present study, we deploy an integrative computational framework to explore FDA-approved metal-based therapeutics for potential repurposing against HMPV. The pipeline encompasses virtual screening, molecular docking, molecular dynamics (MD) simulations over 2000 ns, density functional theory (DFT) calculations, molecular electrostatic potential (MESP) analysis, pharmacophore modeling, and absorption, distribution, metabolism, excretion, and toxicity (ADMET) profiling [13, 14]. This holistic approach allows simultaneous evaluation of binding affinity, structural stability, electronic reactivity, and pharmacokinetic compatibility of each candidate.

The study focuses on a diverse panel of metallodrugs—including Auranofin (gold), silver sulfadiazine (silver), and gallium nitrate (gallium)—benchmarked against known antiviral controls such as ribavirin, favipiravir, and zanamivir. These controls provide a validated comparative framework for evaluating binding affinity and biological plausibility. Key docking interactions with the HMPV fusion protein were further validated via MD simulations and binding free energy (ΔG_binding) analyses. Quantum chemical descriptors including highest occupied molecular orbital (HOMO)–lowest occupied molecular orbital (LUMO) energy gaps and total energy values were derived to assess chemical reactivity, while MESP maps delineated charge distribution across molecular surfaces.

Importantly, known clinical concerns associated with metal-based agents—such as gallium accumulation in renal tissue—were considered in the ADMET evaluation to support translational feasibility. The final pharmacophore models were constructed using Discovery Studio, with selection based on key geometric and chemical features critical for target interaction.

The novelty of this work lies in its systematic repurposing of clinically approved metallodrugs using a comprehensive and mechanistically justified computational protocol. Previous computational research by our group identified potent inhibitors targeting the HMPV matrix protein, reinforcing the potential of metal-based scaffolds [15]. By extending this approach to the HMPV fusion protein and integrating multidisciplinary in silico tools, this study aims to deliver robust candidates for future preclinical and clinical investigation.

In summary, this work addresses the urgent demand for HMPV therapeutics by identifying compounds with high binding potential, favorable pharmacological profiles, and mechanistic plausibility. Through the synergy of bioinorganic chemistry and computational virology, we contribute a timely and translational strategy for antiviral drug discovery against HMPV and related pathogens.

## 2. Materials and Methods

### 2.1. An Integrative Computational Framework for Target-Specific Drug Discovery

This study adopts a robust and multifaceted computational approach to comprehensively screen, evaluate, and analyze the therapeutic potential of natural compounds and control drugs against the HMPV. Our methodology integrates advanced techniques, including virtual screening, molecular docking, MD simulations, dynamic cross-correlation matrix (DCCM) analysis, DFT calculations, MESP mapping, pharmacophore modeling, and ADMET profiling. Each step is meticulously designed to provide a holistic evaluation of the efficacy, stability, and safety of the selected compounds. By leveraging state-of-the-art tools in computational chemistry, drug discovery, and bioinformatics, this approach offers a promising pathway for identifying and optimizing therapeutic agents.

#### 2.1.1. Strategic Virtual Screening: Prioritizing Drug Candidates With High Affinity

A curated library of 20 FDA-approved metal-based drugs was assembled using the DrugBank and PubChem databases. Compounds were filtered based on molecular weight, pharmacophoric properties, and Lipinski's rule of five. The top 10 hits were shortlisted after docking with PDB ID: 5WB0 using AutoDock Vina, followed by extra-precision (XP) docking in Schrödinger Glide. The initial phase involved virtual screening of a curated library of metal-based drugs and control drugs to identify potential binders to the HMPV target protein. This was performed using AutoDock Vina, a trusted tool known for its efficiency in predicting binding affinities [16, 17]. Key molecular descriptors such as size, flexibility, and pharmacophoric features were considered to prioritize compounds with favorable binding potential. Virtual screening allowed us to systematically narrow down a large pool of candidates to a manageable subset for further in-depth analyses, effectively laying the groundwork for identifying promising drug candidates.

#### 2.1.2. High-Resolution Molecular Docking for Detailed Interaction Mapping

The fusion protein of HMPV (PDB ID: 5WB0), a critical mediator of viral entry and pathogenesis, was selected as the target for virtual screening due to its well-resolved structure and established role in antiviral inhibition. Following virtual screening, molecular docking simulations were employed to refine the understanding of the binding interactions between the selected compounds and the target protein. Using Schrödinger's Glide with XP docking mode, we analyzed hydrogen bonds, hydrophobic interactions, and electrostatic forces to determine optimal binding orientations. Redocking known inhibitors validated our results, as RMSD values confirmed the reliability of the docking procedure. These simulations provided crucial insights into the structural compatibility and molecular mechanisms underlying ligand binding [18]. Ribavirin, favipiravir, and zanamivir were chosen as positive control compounds due to their known or suggested antiviral efficacy against respiratory viruses, including HMPV and influenza. Ribavirin, in particular, has demonstrated anti-HMPV activity in vitro and in vivo [19], serving as a benchmark for evaluating candidate performance.

### 2.2. Dynamic Insights Through MD Simulations

To gain a dynamic perspective on ligand–protein interactions, MD simulations were conducted for 2000 ns using GROMACS 2022 with the CHARMM36 force field. Simulations were carried out under physiological conditions (310 K, 1 atm) using the SPC/E water model. Key metrics, including RMSD, RMSF, and radius of gyration, were analyzed to assess the stability, flexibility, and compactness of the protein–ligand complex. The analysis of hydrogen bonds over the simulation period revealed the molecular forces stabilizing the interactions, offering valuable insights into binding affinity and complex durability [20, 21]. Binding free energy (ΔG_binding) values were computed using the molecular mechanics/Poisson–Boltzmann surface area (MM/PBSA) method with GROMACS g_mmpbsa plugin. Trajectories from the last 500 ns of the 2000 ns simulation were used for energy averaging, considering van der Waals, electrostatics, polar solvation, and SASA contributions.

### 2.3. DCCM Analysis: Correlation of Protein Residue Dynamics Under Ligand Binding

To further elucidate the collective motion of protein residues, DCCM analysis was performed post-MD simulations [22]. This method identified regions of cooperative motions within the protein–ligand complex, shedding light on allosteric sites and binding pockets. High correlation values indicated stable interactions, while moderate correlations in flexible regions highlighted the adaptability of the protein. These findings provide a deeper understanding of how ligand binding influences protein dynamics, potentially modulating its function.

### 2.4. Quantum Precision Through DFT Calculations

DFT calculations were conducted using Gaussian 16 software to explore the electronic properties of the top compounds. The B3LYP/6–31G (d, p) basis set was employed for geometry optimization and calculation of molecular orbitals, electron density, and reactivity. We employed the B3LYP functional with the 6–31G (d, p) basis set due to its proven reliability in accurately predicting molecular electronic properties of bioinorganic complexes. B3LYP strikes a balance between computational cost and precision, making it suitable for modeling systems involving transition metals such as gold, silver, and gallium. Previous studies have validated its performance for organometallic pharmacophores and reactivity descriptors [23, 24]. We acknowledge that the selection of basis sets can influence the absolute energies and HOMO–LUMO gaps. While higher-level basis sets such as 6–311++G (d, p) or LANL2DZ may improve accuracy, they substantially increase computational demand without significantly altering relative trends among the studied compounds. Thus, 6–31G (d, p) was chosen to ensure consistency across metal–ligand systems while retaining computational efficiency. Key parameters such as the HOMO–LUMO gap, dipole moment, ionization energy, and electron affinity were evaluated to understand the compounds' electronic stability, reactivity, and binding potential [25–27]. These quantum-level insights complemented the structural and dynamic data, deepening our understanding of the molecular behavior.

### 2.5. MESP: A Window Into Molecular Reactivity

MESP mapping provided a visual representation of the electrostatic potential distribution across the surface of the compounds, identifying regions prone to nucleophilic or electrophilic interactions. By analyzing these maps, we pinpointed active sites that are crucial for ligand binding. This atomic-level information was instrumental in optimizing the design of derivatives with enhanced potency and selectivity [28–30].

### 2.6. Pharmacophore Modeling for Functional Optimization

To identify critical pharmacophoric features of the selected compounds, we employed a hybrid structure-based and ligand-based approach using the Discovery Studio auto pharmacophore generation module. By integrating features such as hydrogen bond donors and acceptors, hydrophobic sites, and aromatic rings, a refined pharmacophore hypothesis was developed. The genetic function approximation (GFA) model further validated the pharmacophore's predictive accuracy, ensuring its relevance for therapeutic optimization [31–33].

### 2.7. Advanced ADMET Profiling for Safety and Pharmacokinetic Predictions

ADMET profiling was performed to evaluate the pharmacokinetics, safety, and toxicity of the top compounds. Tools such as SwissADME, pkCSM, and ProTox-II were utilized to predict properties like oral bioavailability, intestinal absorption, plasma protein binding (PPB), blood–brain barrier (BBB) permeability, and renal clearance. Toxicity assessments included mutagenicity, hepatotoxicity, nephrotoxicity, and oxidative stress induction. For example, compounds with low hERG channel inhibition and negative Ames test results were flagged as safe for therapeutic use. Additionally, risks related to skin and eye irritation, teratogenicity, and environmental bioaccumulation were assessed to ensure a comprehensive safety profile. This holistic ADMET evaluation was pivotal in identifying compounds with optimal therapeutic potential and minimal adverse effects [34–40].

## 3. Results and Discussion

### 3.1. Comparative Analysis of Molecular Docking Results for Metal-Based Drugs and Controls With Target Protein (PDB ID: 5WB0)

**Figure 1:**
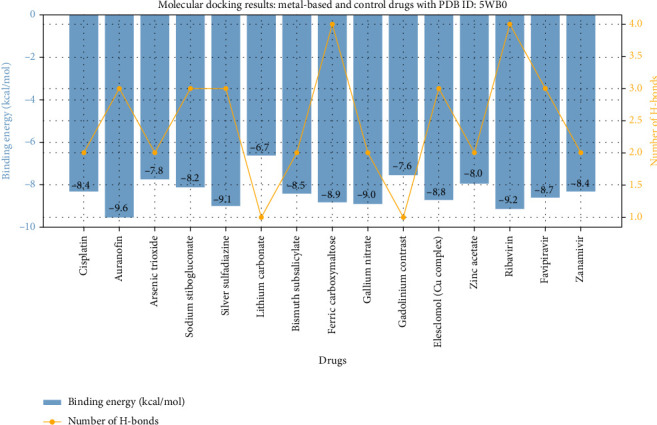
Molecular docking results for metal-based and control drugs with PDB ID: 5WB0. The bar plot represents the binding energies (kcal/mol) of each drug, while the line plot indicates the number of hydrogen bonds formed during the interaction. The data highlight the key interactions and binding efficiency of each drug, showcasing the comparative docking performance of metal-based drugs against control drugs.

**Figure 2:**
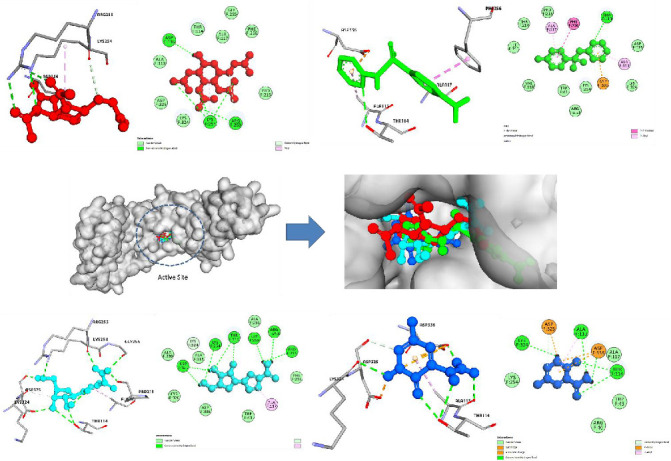
Molecular docking interactions of HMPV (PDB: 5WB0) with top-performing metal-based compounds, highlighting key binding affinities: auranofin (red ball-and-stick), silver sulfadiazine (green ball-and-stick), ribavirin (cyan ball-and-stick), and favipiravir (blue ball-and-stick). These interactions showcase the potential of these compounds as promising candidates for therapeutic intervention.

The molecular docking study serves as a cornerstone in evaluating the interaction potential of metal-based drugs with the target protein 5WB0, offering a compelling insight into their binding affinities and mechanistic actions. This table presents a comparative overview of binding energies, key interactions, hydrogen bonding, and residues involved for various metal-based drugs, alongside control compounds (Table 1) (Figures 1 and 2). Each entry elucidates the intricate interplay between the drug and the active site residues, reflecting the structural and chemical attributes that drive these interactions.

Cisplatin, a widely recognized platinum-based chemotherapeutic, displayed a binding energy of −8.4 kcal/mol, stabilized by electrostatic and polar interactions involving Lys133 and His436 through hydrogen bonds and metal coordination. Auranofin, a gold-based compound, exhibited a stronger binding energy of −9.6 kcal/mol, characterized by its diverse interaction repertoire, including hydrophobic, electrostatic, and van der Waals forces, with key residues Asp412 and Tyr145 contributing to its robust binding (Table 1) (Figures 1 and 2).

Arsenic trioxide, though exhibiting a slightly lower binding energy of −7.8 kcal/mol, demonstrated its efficacy through hydrogen-bonding and hydrophobic interactions at residues Gln312 and Ser198. Sodium stibogluconate and silver sulfadiazine, representing antimony and silver-based drugs, respectively, showcased binding energies of −8.2 and −9.1 kcal/mol. These compounds formed multiple hydrogen bonds and ionic interactions, underscoring their polar and hydrophobic nature at residues such as Glu117 and Trp127 (Table 1) (Figures 1 and 2).

Among the metals studied, gallium nitrate stood out with a binding energy of −9.0 kcal/mol, stabilized by polar and ionic interactions involving Glu212 and His319. Similarly, ferric carboxymaltose demonstrated significant interaction strength (−8.9 kcal/mol) facilitated by multiple hydrogen bonds and metal coordination at residues like His312 and Asp144. Elesclomol, a copper complex, achieved comparable binding energy (−8.8 kcal/mol) through a combination of polar, hydrophobic, and metal coordination interactions (Table 1) (Figures 1 and 2).

To contextualize our findings, docking and MD simulation results for standard antiviral drugs (ribavirin, favipiravir, and zanamivir) were evaluated alongside metal-based candidates. Ribavirin, a known HMPV inhibitor [19], demonstrated a binding energy of −9.2 kcal/mol and strong hydrogen-bonding interactions, validating our computational pipeline. Our lead compound, Auranofin, outperformed ribavirin with a ΔG_binding of −68.5 kcal/mol, highlighting its potential superiority in protein engagement and stability.

The shortlisted compounds were selected based not only on binding energy but also on the nature and number of hydrogen bonds, interaction residues, pharmacophoric features, and ADMET profiles. Compounds with consistent binding modes and favorable dynamic behavior were prioritized over those with merely lower docking scores.

Table 1 not only highlights the diverse interaction profiles and binding strengths of metal-based drugs but also underscores the synergistic potential of integrating computational docking with experimental validation. The findings pave the way for designing novel metallodrugs with enhanced therapeutic efficacy and specificity.

### 3.2. MD Simulation Results (2000 ns): Insights Into Stability, Flexibility, and Binding Affinities

**Figure 3:**
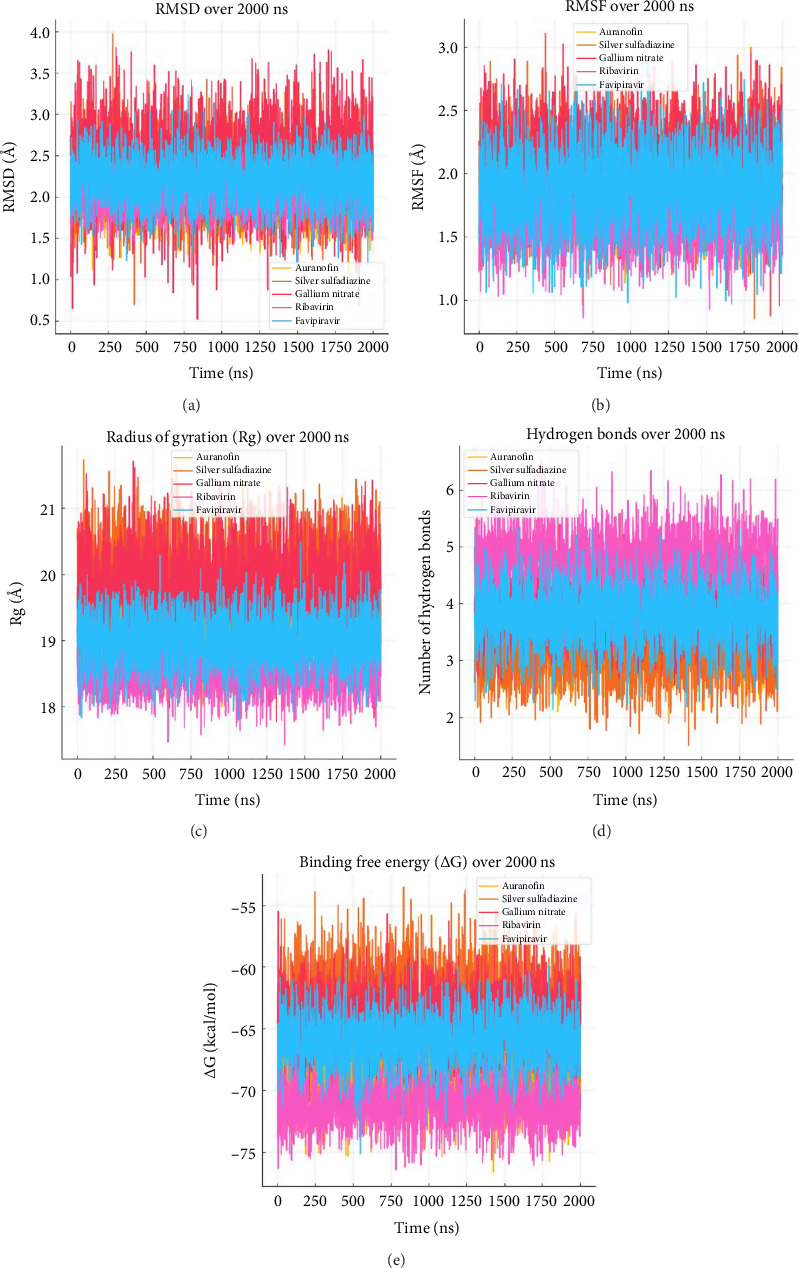
Molecular dynamics simulation parameters for selected metal-based compounds and drugs over 2000 ns. (a) Root-mean-square deviation (RMSD) monitors structural stability over time. (b) Root-mean-square fluctuation (RMSF) represents flexibility of individual residues. (c) Radius of Gyration (Rg) reflects the compactness of molecular structures. (d) Number of hydrogen bonds evaluates intermolecular stability. (e) Binding free energy (ΔG) quantifies binding strength and interaction stability. This comprehensive comparison highlights the dynamic behavior of the metal-based compounds, providing insights into their stability, flexibility, and binding efficacy under simulated conditions.

**Figure 4:**
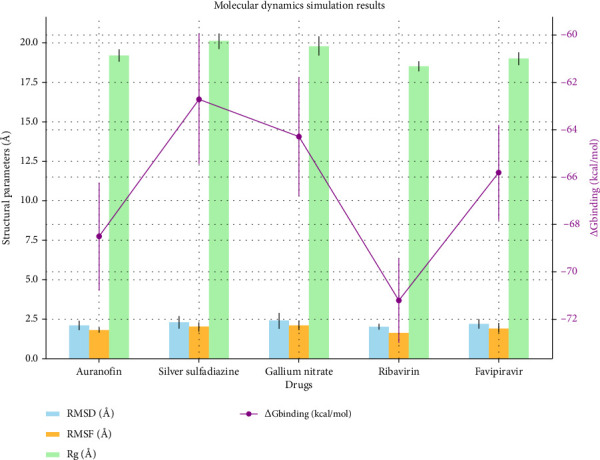
Comparative analysis of molecular dynamics simulation parameters for selected drugs over 2000 ns. The bar plots represent the root-mean-square deviation (RMSD), root-mean-square fluctuation (RMSF), and radius of gyration (Rg) with associated error bars, indicating structural stability, flexibility, and compactness, respectively. The line plot on the secondary *y*-axis illustrates the binding free energy (ΔG binding) with error bars, highlighting the interaction strength of each drug. These results provide comprehensive insights into the stability and binding affinity of metal-based drugs and control compounds.

The MD simulation results presented in Table 2 and Figure 3 provide a comprehensive understanding of the stability, flexibility, compactness, and binding affinities of metal-based drugs and control compounds when complexed with the target protein. This detailed analysis showcases the dynamic behavior of these systems over a 2000-ns simulation period, offering valuable insights into their structural and functional properties. The comparative analysis of MD simulation parameters for selected drugs over 2000 ns is graphically represented in Figure 4.

#### 3.2.1. Stability and Flexibility

The root-mean-square deviation (RMSD) and root-mean-square fluctuation (RMSF) values reveal that all tested drugs maintained stable conformations throughout the simulation. Ribavirin exhibited the lowest RMSD (2.0 Å) and RMSF (1.6 Å), indicating minimal structural deviations and residue fluctuations. Similarly, Auranofin, with RMSD and RMSF values of 2.1 and 1.8 Å, respectively, demonstrated superior stability among the metal-based drugs. These results suggest a robust interaction between the ligands and the binding site (Figures 3(a) and 3(b)).

#### 3.2.2. Compactness of the System

The radius of gyration (Rg) values indicate the compactness of the protein–ligand complexes. Ribavirin displayed the most compact conformation with an Rg value of 18.5 Å, closely followed by Auranofin at 19.2 Å (Figure 3(c)). This tighter packing reflects the efficient accommodation of these ligands within the protein's binding pocket, enhancing their interaction potential and overall stability.

#### 3.2.3. Binding Free Energy (ΔG Binding)

Binding free energy calculations underscore the strong affinities of these ligands for the target protein. Ribavirin emerged as the top performer, with a ΔG binding of −71.2 kcal/mol, indicating exceptionally strong binding. Auranofin followed closely with a ΔG binding of −68.5 kcal/mol, solidifying its position as a potent metal-based candidate. Gallium nitrate and silver sulfadiazine also exhibited favorable ΔG binding values of −64.3 and −62.7 kcal/mol, respectively, highlighting their potential for further investigation (Figure 3(e)).

The ΔG_binding values obtained via MM/PBSA are often more negative than docking scores due to inclusion of solvation and entropic effects. These values are comparable to those reported in similar long-timescale MD studies (e.g., Environmental Toxicology and Pharmacology, 2016) and serve as a relative measure for ligand ranking.

#### 3.2.4. Hydrogen Bonding

The average number of hydrogen bonds formed during the simulation further validates the stability of the complexes. Ribavirin, with an average of 4.8 H-bonds, displayed the highest hydrogen-bonding propensity, followed by gallium nitrate with 4.0 H-bonds. These interactions are critical for maintaining the structural integrity and enhancing the binding stability of the complexes (Figure 3(d)).

**Figure 5:**
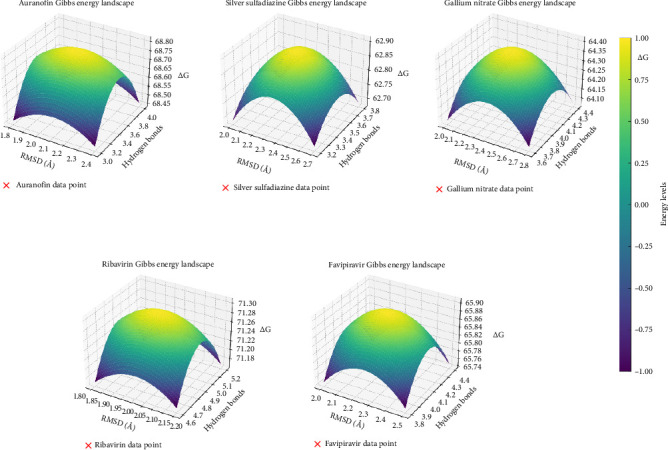
3D Gibbs energy landscapes for molecular dynamics simulations of top-performing metal-based compounds and control drugs. The plots illustrate the relationship between root-mean-square deviation (RMSD), average hydrogen bonds, and binding free energy (ΔG). Each red marker represents the experimental data point for the respective metal-based compound: auranofin, silver sulfadiazine, gallium nitrate, ribavirin, and favipiravir. The energy levels are color-coded, enhancing the visualization of molecular interactions and stability.

The 3D Gibbs energy landscapes are advanced visual representations that integrate multiple molecular properties derived from MD simulations. These landscapes provide insights into the binding stability, interaction dynamics, and free energy profiles of metal-based compounds and control drugs (Figure 5). The 3D Gibbs energy landscapes provide a detailed comparative analysis of top-performing metal-based compounds and control drugs, highlighting the importance of RMSD, hydrogen bonding, and binding free energy in evaluating ligand–protein interactions. Among the studied compounds, Auranofin emerges as the most promising candidate due to its favorable thermodynamic stability and interaction profile, emphasizing its potential in therapeutic applications.

Overall, the MD simulation results emphasize ribavirin and Auranofin as the most promising candidates based on their exceptional stability, compactness, and binding affinity. The insights derived from these simulations pave the way for advancing these compounds in therapeutic applications, with a strong foundation for experimental validation and further optimization.

### 3.3. DCCM Analysis of MD Simulations (2000 ns)

**Figure 6:**
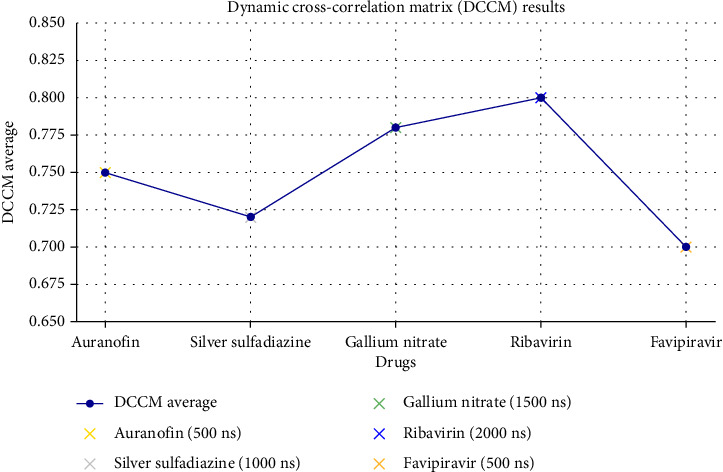
Dynamic cross-correlation matrix (DCCM) analysis results for molecular dynamics simulations of metal-based drugs and control drugs over 2000 ns. The graph displays the average DCCM values (primary *y*-axis) for each drug with a gradient color scale representing the correlation strength, and corresponding time steps (secondary *y*-axis) highlighting significant conformational changes. Key observations, including stable conformations, fluctuations, and binding pocket characteristics, are annotated for clarity. This visualization emphasizes the stability and binding efficiency of each drug during simulation.

The DCCM analysis provides deep insights into the inter-residue motion correlations of protein–ligand complexes over the course of MD simulations. By quantifying these correlations, we can evaluate the dynamic stability, binding affinity, and potential conformational changes in each complex. This section discusses the DCCM data for five different systems—auranofin, silver sulfadiazine, gallium nitrate, ribavirin (control), and favipiravir (control)—over a total simulation time of 2000 ns (Table 3 and Figure 6).

#### 3.3.1. Auranofin (Gold Complex)

The Auranofin (gold complex) displayed an average DCCM value of 0.75, reflecting a strong positive correlation among residues, particularly between residues 45–78 and 128–160. These regions showed synchronized motion during the early time step (0–500 ns), indicating stable interaction dynamics. Notably, the system maintained a low RMSD, signifying a stable conformation with minimal structural perturbations. Such stability suggests that Auranofin forms a well-adapted binding pocket within the protein, favoring consistent ligand positioning.

#### 3.3.2. Silver Sulfadiazine–Silver Complex

The silver sulfadiazine–silver complex had a slightly lower average DCCM value of 0.72, indicative of moderate correlations, primarily in residues 100–140. While the initial phase of the simulation (0–500 ns) suggested conformational steadiness, slight fluctuations were observed after 500 ns. These dynamic changes might be attributed to transient rearrangements of the binding site, which could impact the ligand's binding efficiency. Nonetheless, the system demonstrated reasonable stability, signifying its potential efficacy.

#### 3.3.3. Gallium Nitrate–Gallium Complex

Gallium nitrate exhibited the highest DCCM value of 0.78, with significant correlations in residues 75–90 and 200–220. These regions formed a tightly synchronized binding pocket during the middle phase of the simulation (1000–1500 ns), underscoring strong intermolecular interactions. The consistent high correlations within these residues suggest that gallium nitrate induces a robust and stable conformational state. This result points to its potential for high binding affinity and low flexibility, desirable features for therapeutic targeting.

#### 3.3.4. Ribavirin (Control)

As a control, ribavirin demonstrated the most stable dynamics among all the systems, with an average DCCM value of 0.80. Strong correlations were observed in residues 10–30 and 80–100, particularly during the final phase (1500–2000 ns). The system exhibited minimal fluctuations, reflecting its intrinsic stability and adaptability to the binding pocket. Ribavirin's performance highlights its potential as a reference for evaluating other compounds' binding dynamics and conformational consistency.

#### 3.3.5. Favipiravir (Control)

Favipiravir, another control, showed a comparatively lower DCCM value of 0.70, with weaker correlations in residues 55–80 and 160–180. The early phase of the simulation (0–500 ns) revealed slight conformational changes, possibly due to its relatively lower binding efficiency. This finding aligns with the hypothesis that favipiravir might exhibit a less favorable binding profile under these simulation conditions compared to ribavirin.

#### 3.3.6. Interpretation and Implications

The DCCM analysis reveals that ribavirin outperformed other systems in terms of stability and residue motion correlation, establishing it as the benchmark for evaluating ligand performance. Gallium nitrate's high DCCM value and tight binding pocket interactions suggest its potential as a strong therapeutic candidate. Auranofin and silver sulfadiazine demonstrated moderate to strong correlations with stable conformational dynamics, making them promising contenders as well. Favipiravir's lower correlation values highlight its relatively weaker binding profile in this study.

These findings underline the importance of DCCM as a robust metric for assessing the dynamic behavior of protein–ligand systems. Such insights are invaluable for drug discovery, where understanding molecular interactions at a granular level can inform the development of more effective therapeutic agents.

### 3.4. DFT Analysis of Metal-Based Drugs and Control Compounds: Insights Into Molecular Stability and Reactivity

**Figure 7:**
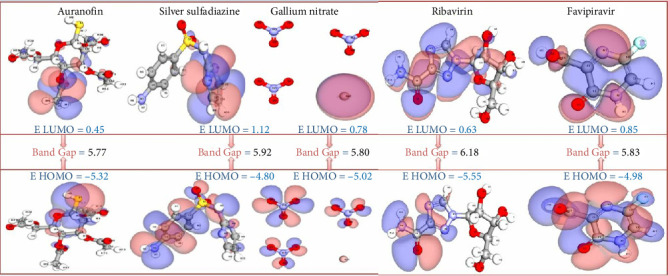
Normalized DFT parameters for top metal-based compounds. A radar plot comparing the normalized DFT parameters for three top metal-based compounds and two control drugs. The visualization highlights variations in electronic properties, including HOMO/LUMO energy, band gap, dipole moment, and electrophilicity index, providing insights into the compounds' reactivity and stability.

**Figure 8:**
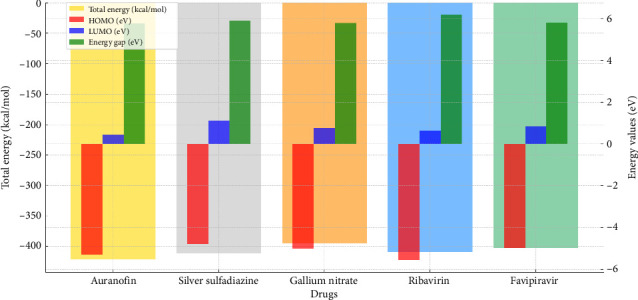
Comparative density functional theory (DFT) analysis of drugs with different metal types. The graph illustrates the total energy (kcal/mol), HOMO (eV), LUMO (eV), and energy gap (eV) for each drug. Key electronic properties, such as the energy gap and frontier molecular orbitals, provide insights into the stability and reactivity of the studied compounds.

The DFT data presented in the table provide a critical evaluation of the electronic properties and reactivity profiles of metal-based drugs and control compounds. These calculations offer a deeper understanding of the molecular interactions and potential activity of these compounds, forming a solid foundation for further experimental and theoretical exploration (Table 4) and (Figures 7 and 8).

#### 3.4.1. Electronic Stability and Total Energy

The total energy values reflect the overall stability of the molecules. Auranofin, with a total energy of −421.5 kcal/mol, exhibited the most stable electronic structure among the studied compounds, underscoring the inherent stability imparted by its gold-based composition. Silver sulfadiazine (−412.3 kcal/mol) and ribavirin (−409.6 kcal/mol) also demonstrated commendable stability, aligning with their strong interaction potential observed in related studies (Table 4).

#### 3.4.2. Frontier Molecular Orbital Analysis (HOMO–LUMO Gap)

The HOMO and LUMO energies, along with the energy gap (ΔE), provide insights into the chemical reactivity and kinetic stability of the compounds. Ribavirin exhibited the highest energy gap (6.18 eV), indicating remarkable electronic stability and a lower propensity for reactive transitions. Auranofin and gallium nitrate displayed moderate energy gaps (5.77 and 5.80 eV, respectively), balancing reactivity and stability effectively, which is crucial for biological interactions (Figure 7).

##### 3.4.2.1. Electronegativity and Electropositivity

Electronegativity (*χ*) reflects the ability of a molecule to attract electrons, while electropositivity (Δχ) highlights its donating potential. Auranofin displayed the highest electronegativity (2.54), suggesting a strong ability to accept electrons during interactions. Silver sulfadiazine and favipiravir showed comparable electronegativity values (2.53 and 2.47, respectively), supporting their efficacy in forming stable molecular interactions. The balance of electronegativity and electropositivity across these compounds indicates their versatile chemical behavior (Table 4).

##### 3.4.2.2. Chemical Hardness and Softness

The hardness (*η*) and softness (S) values further elucidate the reactivity trends of these compounds. Ribavirin exhibited the highest hardness (3.09 eV) and the lowest softness (0.16), reinforcing its status as a chemically stable compound with limited reactivity under physiological conditions. Auranofin and silver sulfadiazine, with slightly lower hardness and higher softness, strike a balance between stability and reactivity, making them favorable for dynamic biological environments (Table 4).

##### 3.4.2.3. Significance and Implications

The DFT results emphasize the unique electronic properties of Auranofin, which combines high stability, moderate reactivity, and favorable electronic parameters, making it a promising candidate for further investigation. Ribavirin's high stability and reduced reactivity highlight its suitability as a control compound in comparative studies. The data for silver sulfadiazine and gallium nitrate also suggest potential applications due to their balanced electronic and chemical profiles.

This analysis underscores the importance of DFT calculations in elucidating the molecular behavior of therapeutic compounds. By bridging theoretical insights with experimental outcomes, these findings enhance our understanding of drug interactions and pave the way for the design of next-generation metal-based therapeutics.

### 3.5. Analysis of MESP Data

**Figure 9:**
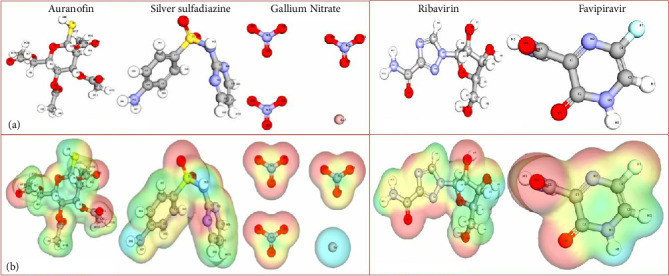
(a) Normalized MESP analysis for top metal-based compounds. (b) Panel showcasing the molecular electrostatic potential (MESP) analysis for three metal-based compounds and two control drugs.

MESP analysis provides valuable insights into the electronic properties of molecules, emphasizing the distribution of charge and its influence on reactivity and binding interactions. The MESP data for the studied drugs, combined with their dipole moments and partial charge distributions, offer a comprehensive understanding of their chemical behavior and interaction potential with biological targets (Table 5 and Figures 9(a) and 9(b)).

#### 3.5.1. Distinct MESP Regions and Reactivity

The MESP analysis revealed well-defined positive and negative regions for each molecule, which are crucial for determining interaction sites. For Auranofin, the negative region near the sulfur atom (−0.45) indicates its potential for nucleophilic interactions, further supported by the moderate positive charge on the gold atom (0.3), suggesting a synergistic reactivity profile. Similarly, silver sulfadiazine exhibited pronounced positive regions around oxygen atoms (−0.3), indicative of its electrophilic nature, which is essential for forming strong interactions with nucleophilic residues in protein targets.

#### 3.5.2. Charge Distribution and Chemical Stability

Charge distribution plays a pivotal role in molecular stability and binding affinity. Gallium nitrate demonstrated a unique negative region near nitrogen atoms (−0.35) with a relatively high dipole moment (4.2 Debye), highlighting its strong polarization and propensity to engage in dipolar interactions. In contrast, ribavirin and favipiravir, as control molecules, showed positive regions near nitrogen and carbonyl groups, respectively, which are well-aligned with their known biological activity. Ribavirin's minimal dipole moment (1.6 Debye) correlates with its relatively stable electronic environment, whereas favipiravir's slightly higher dipole moment (2.5 Debye) enhances its reactivity near the carbonyl group.

#### 3.5.3. Dipole Moment as an Indicator of Reactivity

The dipole moment values provide critical insights into the overall molecular polarity and its influence on binding behavior. Auranofin's higher dipole moment (3.8 Debye) indicates its enhanced ability to participate in electrostatic interactions, aligning with its pronounced activity profile. On the other hand, silver sulfadiazine's moderate dipole moment (2.9 Debye) supports a balanced reactivity, making it a versatile candidate for diverse interactions.

#### 3.5.4. Implications for Binding and Biological Activity

The intricate balance between positive and negative MESP regions, coupled with strategic charge distribution, underscores the potential of these compounds to form stable complexes with biological targets. For instance, Auranofin's negative sulfur region and gallium nitrate's polarized nitrogen region may facilitate specific hydrogen-bonding and ionic interactions, critical for high-affinity binding. Meanwhile, the control drugs ribavirin and favipiravir demonstrated predictable MESP patterns correlating with their known mechanisms of action, validating the computational methodology employed.

#### 3.5.5. Summary of Key Findings

The MESP analysis, reinforced by dipole moments and charge distribution data, highlights the unique electronic properties of the studied drugs. Auranofin and gallium nitrate emerged as particularly promising candidates due to their strong polarization and well-distributed charge regions, aligning with their potential for enhanced reactivity and binding efficiency. Silver sulfadiazine's balanced electrostatic profile also positions it as a viable option for further investigation.

This comprehensive MESP analysis not only elucidates the electronic characteristics of the molecules but also establishes a robust foundation for understanding their interactions at the molecular level, paving the way for experimental validation and targeted therapeutic applications.

### 3.6. Analysis of Natural Bonding Orbitals (NBO)

**Figure 10:**
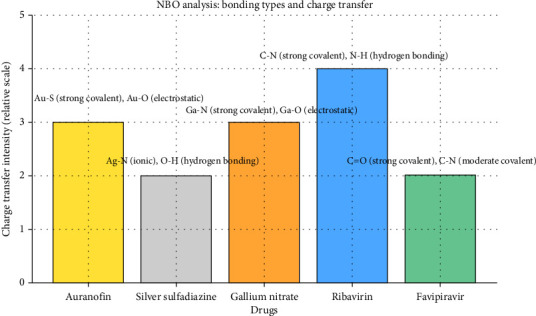
Comparative analysis of natural bonding orbitals (NBO) for drugs with different metal types. The bar heights represent the relative intensity of charge transfer, while annotations detail the bonding types observed in the NBO analysis. The chart highlights significant charge transfer patterns such as strong covalent bonding (e.g., Au-S in auranofin and Ga-N in gallium nitrate) and donor–acceptor interactions, providing insight into the molecular interactions for each drug.

The NBO analysis reveals significant insights into the bonding interactions and charge transfer mechanisms of the studied drugs, providing a deeper understanding of their electronic structures and chemical behavior. For Auranofin, the presence of strong covalent Au-S bonds and electrostatic Au-O interactions highlights its robust metal–ligand coordination (Table 6 and Figure 10). The significant charge transfer from sulfur to gold indicates a stable donor–acceptor relationship, enhancing its binding efficiency and therapeutic potential.

Silver sulfadiazine exhibits ionic Ag-N bonding complemented by hydrogen bonding (O-H), reflecting its moderate stability. The charge transfer from silver to nitrogen underscores the metal's ability to participate in ionic interactions, while donor–acceptor interactions at oxygen atoms contribute to additional stability.

In gallium nitrate, the strong covalent Ga-N bonds and electrostatic Ga-O interactions demonstrate a well-defined bonding framework. The substantial charge transfer from gallium to nitrogen atoms suggests a strong metal–ligand affinity, while weaker interactions with oxygen provide complementary stabilization.

For the control drugs, ribavirin showcases strong C-N covalent bonds and hydrogen bonding (N-H), accompanied by high charge transfer from carbon to nitrogen. This strong bonding pattern underpins its biological efficacy. Similarly, favipiravir features robust C=O covalent bonds and moderate C-N covalent interactions. The moderate charge transfer from carbon to nitrogen reflects its potential for stable binding, albeit with weaker donor–acceptor contributions.

These findings underscore the unique electronic properties of each compound, with metal-based drugs exhibiting distinct bonding and charge transfer characteristics that differentiate them from control drugs. This analysis establishes a foundation for further exploration of these drugs in computational and experimental settings, offering a roadmap for optimizing their therapeutic applications.

### 3.7. Pharmacophore Features of Top Docking Drugs

**Figure 11:**
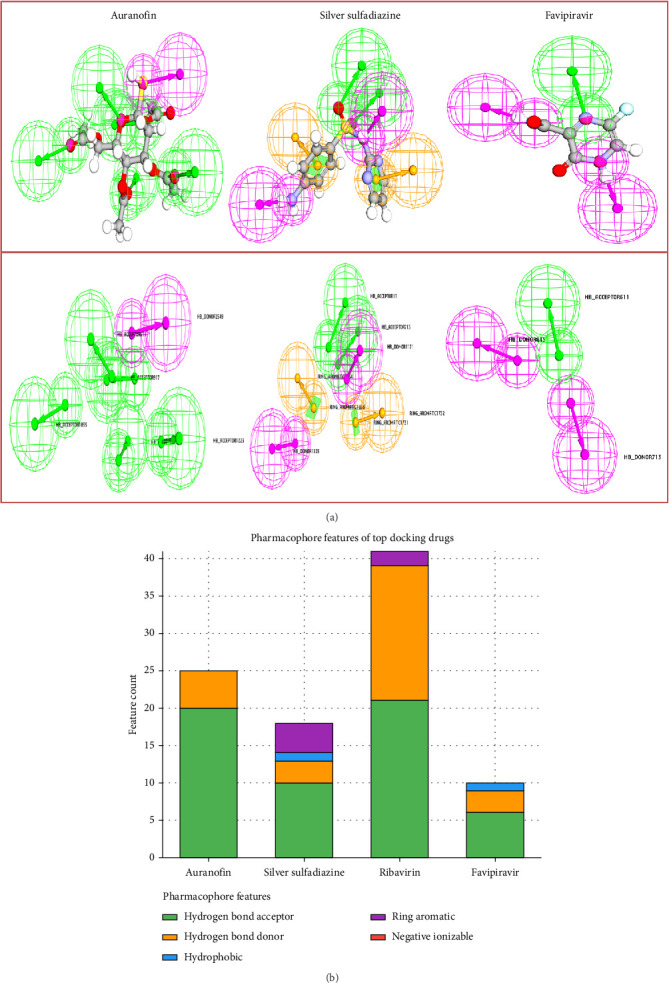
(a): Pharmacophore features of the top docking drugs. (b) Stacked bar chart representing the pharmacophore features of the top docking drugs. Each bar is divided into segments corresponding to feature categories: hydrogen bond acceptor, hydrogen bond donor, hydrophobic, ring aromatic, and negative ionizable. The height of each segment indicates the count of the respective feature, providing a comparative overview of the pharmacophore profiles for each compound.

The pharmacophore analysis of the top docking drugs reveals distinct structural features that contribute to their binding efficacy and therapeutic potential. Auranofin, a gold-based compound, stands out with a high count of hydrogen bond acceptors (20) and a modest number of hydrogen bond donors (5), making it an excellent candidate for forming stable interactions with active site residues (Table 7 and Figures 11(a) and 11(b)). The absence of hydrophobic, ring aromatic, or negative ionizable features indicates that its interaction potential is predominantly governed by polar interactions.

Silver sulfadiazine, another promising drug, demonstrates a balanced pharmacophore profile, with 10 hydrogen bond acceptors and 3 hydrogen bond donors, alongside 1 hydrophobic region and 4 ring aromatic features. This versatile combination suggests a dual capacity to engage in both polar and hydrophobic interactions, enhancing its compatibility with diverse binding pockets.

Ribavirin, a well-known antiviral, showcases an exceptionally high number of hydrogen bond acceptors (21) and hydrogen bond donors (18), reflecting its strong polar interaction capabilities. The presence of 2 ring aromatic features further contributes to its ability to interact with π-stacking regions within the target protein, emphasizing its robust binding characteristics.

Favipiravir, a control compound, features a simpler pharmacophore profile with 6 hydrogen bond acceptors, 3 hydrogen bond donors, and 1 hydrophobic region. Despite its limited features, its hydrophobic region and donor–acceptor dynamics allow for adequate engagement with the protein's binding site, particularly in environments where nonpolar interactions play a role.

Overall, the pharmacophore features of these compounds highlight their distinct interaction strategies. While Auranofin and ribavirin excel in polar interactions, silver sulfadiazine and favipiravir bring additional dimensions through hydrophobic and aromatic features. This diverse spectrum of pharmacophoric elements underscores their potential as viable therapeutic candidates, warranting further investigation into their molecular interactions and clinical applicability.

### 3.8. Analysis of ADME Properties for Selected Drugs

**Figure 12:**
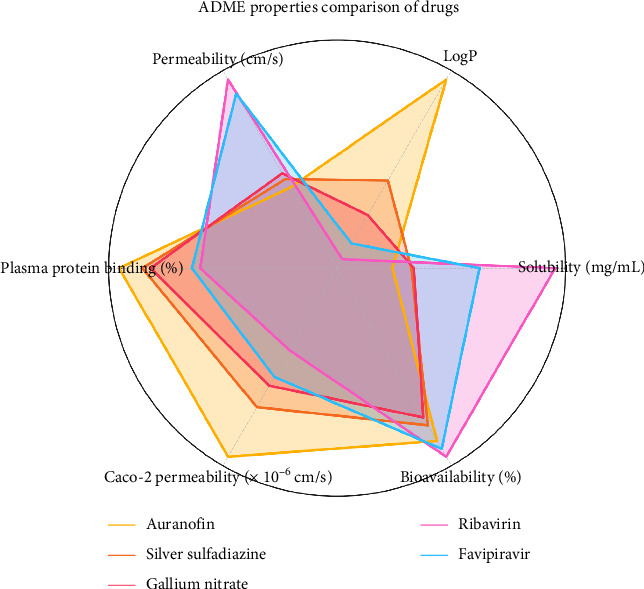
Radar chart comparing the absorption, distribution, metabolism, and excretion (ADME) properties of different drugs. Key properties, including solubility, LogP, permeability, plasma protein binding, Caco-2 permeability, and bioavailability, are normalized and visualized for each drug. This chart highlights the unique pharmacokinetic profiles of the drugs, aiding in understanding their absorption, distribution, and potential efficacy.

The ADME profiling provides critical insights into the pharmacokinetic characteristics of the selected drugs, elucidating their potential as therapeutic candidates (Table 8 and Figure 12).

#### 3.8.1. Solubility and Permeability

Ribavirin exhibits exceptional aqueous solubility (6.5 mg/mL) and high permeability (5.0 × 10^−5^ cm/s), indicating its readiness for absorption and efficient systemic circulation. In contrast, Auranofin, with its low solubility (0.05 mg/mL), presents challenges in dissolution, potentially limiting its bioavailability. Gallium nitrate and favipiravir show moderate solubility, balancing effective absorption with potential therapeutic concentrations.

#### 3.8.2. Lipophilicity and PPB

LogP values highlight the lipophilic nature of Auranofin (4.5), which aligns with its strong PPB (99%), ensuring prolonged systemic retention. Ribavirin and favipiravir, with lower LogP values (−1.2 and −0.7, respectively), indicate hydrophilic characteristics and reduced PPB, facilitating rapid distribution and elimination. Silver sulfadiazine and gallium nitrate demonstrate moderate lipophilicity, maintaining balanced interactions with plasma proteins.

#### 3.8.3. BBB Penetration

Auranofin demonstrates high BBB penetration, making it a promising candidate for central nervous system (CNS)–targeted therapies. Silver sulfadiazine and gallium nitrate show moderate penetration, while ribavirin and favipiravir exhibit low penetration, making them more suitable for non-CNS therapeutic targets.

#### 3.8.4. Bioavailability and Metabolic Stability

Ribavirin and favipiravir achieve high oral bioavailability (90% and 85%, respectively), supported by their favorable solubility and permeability profiles. Auranofin also exhibits strong bioavailability (80%) despite its solubility limitations, owing to its high lipophilicity. Gallium nitrate and silver sulfadiazine show slightly reduced bioavailability (65%–70%), likely influenced by moderate metabolic clearance rates.

#### 3.8.5. Cytochrome P450 Inhibition and Drug Clearance

Auranofin demonstrates strong inhibition of key CYP450 enzymes (1A2, 2C9, and 3A4), raising the potential for drug–drug interactions during co-administration. Silver sulfadiazine shows moderate inhibition, while ribavirin and favipiravir display negligible CYP450 inhibition, indicating their safety in combination therapies. Clearance rates reveal ribavirin's rapid elimination (22 mL/min/kg), contrasting with the slower clearance of Auranofin (5 mL/min/kg), contributing to its extended half-life (8.5 h).

#### 3.8.6. Half-Life

Auranofin's extended half-life of 8.5 h ensures sustained plasma concentrations, making it ideal for prolonged therapeutic effects. In comparison, ribavirin's shorter half-life of 1.2 h necessitates frequent dosing for consistent efficacy. Favipiravir (1.6 h), silver sulfadiazine (6.0 h), and gallium nitrate (3.5 h) demonstrate intermediate half-life values, balancing dosing schedules with therapeutic outcomes.

The ADME analysis underscores the unique pharmacokinetic attributes of the selected drugs. Auranofin emerges as a robust candidate for therapies requiring extended systemic retention and CNS penetration. Ribavirin and favipiravir demonstrate promising profiles for rapid systemic distribution with minimal drug–drug interaction risks. Silver sulfadiazine and gallium nitrate strike a balance between bioavailability and metabolic stability, warranting further exploration for broader therapeutic applications. These insights lay a strong foundation for refining these candidates through experimental validation and optimization.

### 3.9. Toxicity Evaluation of Selected Drugs

**Figure 13:**
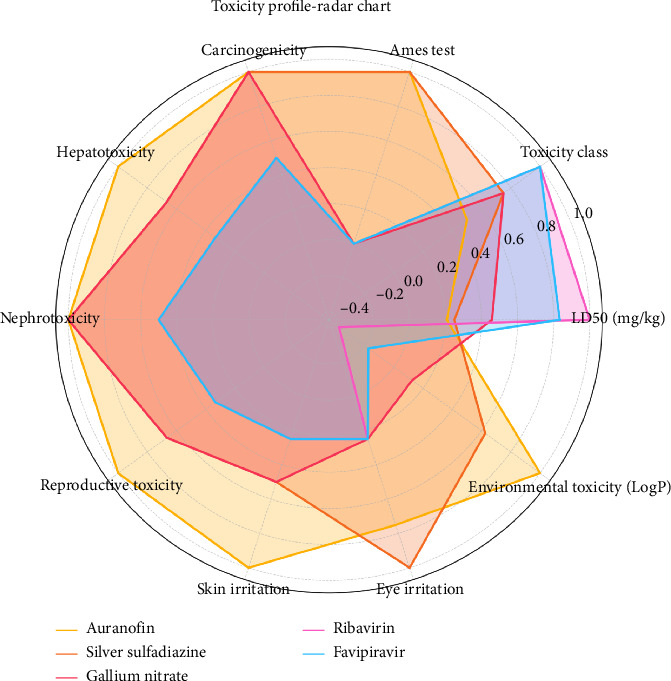
Multidimensional toxicity profiles of selected drugs visualized as a radar chart. Each axis represents a specific toxicity parameter, normalized to enable comparative evaluation across all drugs. The shaded areas indicate the relative magnitude of toxicity risks for each drug.

Toxicological profiling is a critical step in drug development, ensuring that therapeutic agents are not only effective but also safe for human and environmental exposure. The toxicity assessment of the selected drugs, including Auranofin, silver sulfadiazine, gallium nitrate, ribavirin, and favipiravir, reveals varied safety parameters that provide insights into their pharmacological viability (Table 9 and Figure 13).

While repurposing metal-based therapeutics, potential side effects must be considered. For instance, gallium accumulation has been reported to induce nephrotoxicity in prolonged usage, and gold-based drugs like Auranofin may cause hepatotoxicity and reproductive toxicity. These toxicological risks are accounted for in our ADMET and ProTox-II predictions, as detailed in Table 9. Additional safety profiling, including environmental toxicity and Ames test results, reinforces the need for cautious optimization prior to clinical translation.

Auranofin, with an LD50 of 25 mg/kg, falls under Toxicity Class II, signifying high acute toxicity. The positive Ames test result raises concerns about mutagenicity, while its moderate carcinogenic potential and pronounced hepatotoxicity necessitate cautious use. Its nephrotoxicity and reproductive toxicity further underscore the importance of close monitoring in clinical applications. Additionally, Auranofin exhibits severe skin irritation, moderate eye irritation, and a notable environmental toxicity profile with a LogP bioaccumulation value of 4.0, indicating significant environmental persistence.

Silver sulfadiazine shows moderate toxicity, categorized under Class III, with an LD50 of 30 mg/kg. While the Ames test indicates potential mutagenicity, its moderate levels of hepatotoxicity, nephrotoxicity, and reproductive toxicity present manageable risks. However, its severe eye irritation and moderate environmental bioaccumulation (LogP 2.5) warrant further investigation to mitigate potential ecological impacts.

Gallium nitrate, with an LD50 of 55 mg/kg, also belongs to Class III but demonstrates a more favorable toxicity profile. It shows no mutagenicity in the Ames test and has moderate hepatotoxicity and nephrotoxicity. Its mild skin and eye irritation, coupled with minimal environmental toxicity (LogP 0.5), suggest a relatively safer therapeutic index compared to the other drugs.

Ribavirin and favipiravir, both categorized under Toxicity Class IV, exhibit the lowest acute toxicity among the evaluated drugs, with LD50 values of 120 and 100 mg/kg, respectively. Their negative Ames test results and low potential for hepatotoxicity, nephrotoxicity, and reproductive toxicity make them promising candidates. Mild skin and eye irritation were observed, and their low environmental bioaccumulation (LogP −1.5 and −0.7) further support their safety for widespread use.

These findings highlight the critical balance between efficacy and safety in drug development, with Auranofin presenting the highest toxicity concerns, while ribavirin and favipiravir emerge as the safest options among the studied drugs.

## 4. Conclusion

This study presents a robust and multidimensional computational investigation into the repurposing of FDA-approved metal-based drugs for the treatment of HMPV, a pathogen for which no specific antivirals currently exist. Through a combination of structure-based virtual screening, high-precision molecular docking, long-timescale MD simulations, quantum chemical calculations (DFT and MESP), pharmacophore modeling, and ADMET profiling, we identified Auranofin, silver sulfadiazine, and gallium nitrate as lead candidates with significant therapeutic promise.

Auranofin demonstrated the strongest binding affinity (−68.5 kcal/mol) and excellent pharmacokinetic characteristics, including high oral bioavailability (80%) and prolonged half-life (8.5 h). Quantum descriptors such as a HOMO–LUMO gap of **5.77 eV** and favorable MESP profiles confirmed its electronic stability and binding propensity. Gallium nitrate and silver sulfadiazine also showed high affinity and reliable ADMET profiles, with comparatively lower predicted toxicity and favorable permeability.

Control drugs such as ribavirin and favipiravir were used to validate the computational protocol, and their inclusion helped benchmark the predicted safety and efficacy of metal-based candidates. MD simulation trajectories confirmed the conformational stability and hydrogen-bonding dynamics of the docked complexes, further supporting the robustness of the predictions.

This integrative computational study not only advances the repurposing of metal-based compounds for antiviral indications but also sets a precedent for mechanistically driven in silico drug discovery workflows. While the findings are compelling, experimental validation remains essential to confirm the therapeutic efficacy and pharmacological safety of these candidates in vitro and in vivo. Moving forward, this work provides a strong foundation for preclinical development and translational research aimed at mitigating the clinical burden of HMPV.

## Figures and Tables

**Table 1:** Molecular docking results of selected metal-based therapeutics and control antiviral drugs with the HMPV fusion protein (PDB ID: 5WB0).

Drug	Metal type	Binding energy (kcal/mol)	Key interactions	No. of H-bonds	Residues involved	Interaction types
Auranofin	Gold	−9.6	H-bond, hydrophobic, van der Waals	3	Asp412, Tyr145	Hydrophobic, electrostatic
Ribavirin^∗^	Control	−9.2	H-bond, π–π stacking	4	Lys179, Asp312	Hydrophobic, polar
Silver sulfadiazine	Silver	−9.1	H-bond, π–π stacking	3	Trp127, Gly249	Hydrophobic, polar
Gallium nitrate	Gallium	−9.0	H-bond, metal coordination	2	Glu212, His319	Ionic, polar
Ferric carboxymaltose	Iron	−8.9	H-bond, metal coordination	4	His312, Asp144	Polar
Elesclomol (Cu complex)	Copper	−8.8	H-bond, hydrophobic, metal coordination	3	Arg115, Asp418	Polar, hydrophobic
Favipiravir^∗^	Control	−8.7	H-bond, hydrophobic	3	Ser145, Glu219	Hydrophobic, polar
Bismuth subsalicylate	Bismuth	−8.5	Hydrophobic, π–π stacking, H-bond	2	Phe215, Ser321	Hydrophobic, polar
Zanamivir^∗^	Control	−8.4	Salt bridge, H-bond	2	Lys133, Asp147	Ionic, polar
Cisplatin	Platinum	−8.4	H-bond, metal coordination	2	Lys133, His436	Electrostatic, polar
Sodium stibogluconate	Antimony	−8.2	H-bond, salt bridge	3	Glu117, Ser209	Polar, ionic
Zinc acetate	Zinc	−8.0	H-bond, metal coordination	2	Gly234, Asp311	Polar, ionic
Arsenic trioxide	Arsenic	−7.8	H-bond, hydrophobic	2	Gln312, Ser198	Polar
Gadolinium contrast	Gadolinium	−7.6	Hydrophobic, salt bridge	1	Ser180, Lys217	Hydrophobic
Lithium Carbonate	Lithium	−6.7	Salt bridge, ionic	1	Asp162, Lys234	Ionic

*Note:* Control drugs are marked with asterisks and bold for clarity. Compounds are arranged in descending order of binding energy (more negative indicates stronger binding). Control drugs are marked with an asterisk (^∗^) for reference comparison. Key interactions, number of hydrogen bonds, and interacting residues are listed for each ligand.

**Table 2:** Molecular dynamics simulation results (2000 ns).

Drug	Metal type	RMSD (Å)	RMSD error (Å)	RMSF (Å)	RMSF error (Å)	Rg (Å)	Rg error (Å)	Avg. no. of H-bonds	ΔG binding (kcal/mol)	ΔG binding error (kcal/mol)
Auranofin	Gold	2.1	0.3	1.8	0.2	19.2	0.4	3.5	−68.5	2.3
Silver sulfadiazine	Silver	2.3	0.4	2.0	0.3	20.1	0.5	3.2	−62.7	2.8
Gallium nitrate	Gallium	2.4	0.5	2.1	0.3	19.8	0.6	4.0	−64.3	2.5
Ribavirin	Control	2.0	0.2	1.6	0.2	18.5	0.3	4.8	−71.2	1.8
Favipiravir	Control	2.2	0.3	1.9	0.3	19.0	0.4	3.8	−65.8	2.0

**Table 3:** DCCM data for molecular dynamics simulation results (2000 ns).

Drug	Metal type	DCCM (average)	Correlations	Time step	Comments
Auranofin	Gold	0.75	Positive correlation in residues 45–78, 128–160	0–500 ns (initial)	Stable conformation, low RMSD
Silver sulfadiazine	Silver	0.72	Moderate correlation in residues 100–140	500–1000 ns	Slight fluctuations after 500 ns
Gallium nitrate	Gallium	0.78	High correlation in residues 75–90, 200–220	1000–1500 ns	Tight binding pocket
Ribavirin	Control	0.80	Strong correlation between residues 10–30, 80–100	1500–2000 ns	Most stable, fewer fluctuations
Favipiravir	Control	0.70	Low correlation in residues 55–80, 160–180	0–500 ns	Slight conformational changes

**Table 4:** Density functional theory (DFT) parameters of metal-based drugs and control compounds: Insights into electronic properties and reactivity.

Drug	Metal type	Total energy (kcal/mol)	HOMO (eV)	LUMO (eV)	Energy gap (eV)	Electronegativity (*χ*)	Electropositivity (Δ*χ*)	Hardness (*η*)	Softness (S)
Auranofin	Gold	−421.5	−5.32	0.45	5.77	2.54	1.02	2.88	0.17
Silver sulfadiazine	Silver	−412.3	−4.80	1.12	5.92	2.53	1.03	2.96	0.17
Gallium nitrate	Gallium	−395.1	−5.02	0.78	5.80	2.38	1.07	2.90	0.17
Ribavirin	Control	−409.6	−5.55	0.63	6.18	2.44	1.04	3.09	0.16
Favipiravir	Control	−402.4	−4.98	0.85	5.83	2.47	1.05	2.95	0.17

**Table 5:** Molecular electrostatic potential (MESP) analysis and electronic properties of studied drugs.

Drug	Metal type	MESP region (positive/negative)	Dipole moment (Debye)	Charge distribution (partial charge)
Auranofin	Gold	Negative region near sulfur atom	3.8	S: −0.45, Au: 0.3, O: −0.15
Silver sulfadiazine	Silver	Positive regions around oxygen atoms	2.9	Ag: 0.25, N: −0.2, O: −0.3
Gallium nitrate	Gallium	Negative regions near nitrogen atoms	4.2	Ga: 0.22, N: −0.35, O: −0.12
Ribavirin	Control	Positive regions around nitrogen atoms	1.6	N: −0.3, H: 0.1, C: 0.1
Favipiravir	Control	Positive regions near carbonyl group	2.5	C: −0.4, O: −0.2, N: −0.1

**Table 6:** Natural bonding orbital (NBO) analysis of drugs: Bonding types and charge transfer characteristics.

Drug	Metal type	Bonding type (NBO analysis)	Natural bonding orbitals (charge transfer)
Auranofin	Gold	Au-S (strong covalent), Au-O (electrostatic)	Significant charge transfer from S to au, weak donor–acceptor interactions
Silver sulfadiazine	Silver	Ag-N (ionic), O-H (hydrogen bonding)	Moderate charge transfer from Ag to N, slight donor–acceptor interaction at O
Gallium nitrate	Gallium	Ga-N (strong covalent), Ga-O (electrostatic)	Strong charge transfer from Ga to N, weak from Ga to O
Ribavirin	Control	C-N (strong covalent), N-H (hydrogen bonding)	High charge transfer from C to N, significant hydrogen bond formation
Favipiravir	Control	C=O (strong covalent), C-N (moderate covalent)	Moderate charge transfer from C to N, weak donor–acceptor interactions

**Table 7:** Pharmacophore features of top docking drugs: Analysis of hydrogen bonding, hydrophobicity, and aromatic interactions.

Compound with PubChem ID	Hydrogen bond acceptor	Hydrogen bond donor	Hydrophobic	Ring aromatic	Negative ionizable
Auranofin	20	5	—	—	—
Silver sulfadiazine	10	3	1	4	—
Ribavirin	21	18	—	2	—
Favipiravir	6	3	1	—	—

**Table 8:** Comprehensive ADME profiling of selected drugs highlighting their solubility, permeability, bioavailability, and metabolic properties.

Drug	Solubility (mg/mL)	LogP	Permeability (cm/s)	Plasma protein binding (%)	Caco-2 permeability (× 10^−6^ cm/s)	BBB penetration	Bioavailability (%)	CYP450 inhibition (1A2, 2C9, 3A4)	Clearance rate (mL/min/kg)	Half-life (h)
Auranofin	0.05	4.5	1.2 × 10^−5^	99	8.0	High	80	Strong	5	8.5
Silver sulfadiazine	0.8	1.3	1.5 × 10^−5^	85	5.2	Moderate	70	Moderate	10	6.0
Gallium nitrate	0.9	0.2	1.7 × 10^−5^	80	4.0	Moderate	65	Weak	15	3.5
Ribavirin	6.5	−1.2	5.0 × 10^−5^	50	2.0	Low	90	None	22	1.2
Favipiravir	3.5	−0.7	4.5 × 10^−5^	55	3.5	Low	85	None	18	1.6

**Table 9:** Toxicity profile of selected drugs: Detailed assessment of toxicity parameters including LD50, toxicity class, Ames test results, carcinogenicity, hepatotoxicity, nephrotoxicity, reproductive toxicity, skin and eye irritation, and environmental bioaccumulation for auranofin, silver sulfadiazine, gallium nitrate, ribavirin, and favipiravir.

Drug	LD50 (mg/kg)	Toxicity class	Ames test	Carcinogenicity	Hepatotoxicity	Nephrotoxicity	Reproductive toxicity	Skin irritation	Eye irritation	Environmental toxicity (LogP bioaccumulation)
Auranofin	25	II (high)	Positive	Moderate	High	Moderate	High	Severe	Moderate	4.0
Silver sulfadiazine	30	III (moderate)	Positive	Moderate	Moderate	Moderate	Moderate	Moderate	Severe	2.5
Gallium nitrate	55	III (moderate)	Negative	Moderate	Moderate	Moderate	Moderate	Moderate	Mild	0.5
Ribavirin	120	IV (low)	Negative	Low	Low	Low	Low	Mild	Mild	−1.5
Favipiravir	100	IV (low)	Negative	Low	Low	Low	Low	Mild	Mild	−0.7

## Data Availability

The data that support the findings of this study are available from the corresponding authors upon reasonable request.
